# Transfemoral Perimembranous Ventricular Septal Defect Device Closure in Infants Weighing ≤ 10 kg

**DOI:** 10.1007/s00246-023-03100-5

**Published:** 2023-01-25

**Authors:** Dhafer Alshahrani, Niall Linnane, Brian McCrossan, Paul Oslizlok, Colin J. McMahon, Kevin P. Walsh, Damien P. Kenny

**Affiliations:** 1 Department of Pediatric Cardiology, Children’s Health Ireland at Crumlin, Dublin 12, Republic of Ireland; 2 Section of Pediatric Cardiology, Department of Cardiac Sciences, Ministry of National Guard Health Affairs, King Abdulaziz Medical City, Riyadh, Saudi Arabia; 3 Department of Pediatric Cardiology, Royal Belfast Hospital of Sick Children, Belfast, UK

**Keywords:** Congenital heart disease, Pediatric intervention, Perimembranous ventricular septal defect, Congenital cardiac surgery

## Abstract

Transcatheter closure of Perimembranous VSDs (PMVSD) remains challenging particularly in infants. The aim of this study is to evaluate the efficacy and safety of transfemoral PMVSD device closure in infants weighing ≤ 10 kg in a single centre. Retrospective review of departmental databases and medical charts to define patient cohort and collect demographic, procedural and follow-up data. Between July 2014 and March 2021, 16 patients underwent attempted transfemoral PMVSD device closure (12 retrograde) at a median age of 11 months (interquartile range [IQR] 9–15.5) and a median weight of 8.3 kg (IQR 7.2–9.5). All patients were either symptomatic, had progressive left heart dilation or had VSD associated valve regurgitation. Median defect size on pre-procedural transoesophageal echocardiography was 6.8 mm (IQR 6–8.5). Median device waist size was 6 mm (IQR 4.5–8). Successful device placement was achieved in 14 patients (88%). One patient developed moderate aortic and tricuspid valve regurgitation upon retrograde and antegrade device deployment, respectively, and subsequently underwent surgical closure. The second patient developed progressive aortic regurgitation (AR) 2 days post procedure, and also underwent surgical removal with no residual AR. There was no cases of device embolization and no femoral arterial compromise. On median follow-up of 40.5 months (IQR 25–64), none of the patients developed complete heart block. Three patients (18.75%) had small residual shunts at latest follow-up which have not required any further intervention. Device closure of PMVSD’s in children weighing ≤ 10 kg is feasible and safe with good procedural success rates. Use of both the antegrade and retrograde approaches may be necessary depending on anatomical variances.

## Introduction

Surgical closure remains the standard approach for perimembranous VSD (PMVSD) closure especially in infants. However, there is a relatively high morbidity as patients require intensive care unit (ICU) admission and often require blood transfusions [1]. These smaller infants also have a higher risk of AV block [1]. In relation to longer-term consequences, a large population follow-up study has suggested surgical closure is associated with a hazard ratio of 13 compared to the general population of developing atrial fibrillation in the third decade of life [2].

Transcatheter device closure of PMVSDs avoids cardiopulmonary bypass and is associated with shorter hospital stay and less morbidity. Initial concerns regarding unacceptable rates of complete heart block with first generation devices appear to have been circumvented with newer device design, suggesting softer devices with less radial forces might exert less compression on the conduction system [3–10]. Indeed, a recent meta-analysis of transcatheter PMVSD closure in over 6,300 patients revealed a complete atrio-ventricular block (cAVB) rate comparable to surgery at 1.1% [11].

Percutaneous closure of PMVSD in children weighing ≤ 10 kg has been reported to be associated with procedural failure, procedure- or device related adverse events with longer fluoroscopy times [12]. It is technically a greater challenge in lower weight patients with smaller femoral vessels and creation of arteriovenous loops in these patients may result in rhythm disturbances and haemodynamic compromise [13].

Few studies dealing with device closure of PMVSD’s have been published on this subgroup of patients with varying approaches including a trans-carotid approach reported to mitigate against the possible hemodynamic impact of an arteriovenous loop [13–15]. The aim of this study was to evaluate the efficacy and safety of transfemoral PMVSD device closure in infants ≤ 10 kg body weight in a national tertiary care congenital cardiac institution.

## Methodology

Retrospective review of all patients ≤ 10 kgs who underwent attempted closure of PMVSD via a transfemoral approach. Patients were recruited from a single tertiary cardiology centre (Children’s Health Ireland at Crumlin, Dublin, Ireland). Patients were identified and data collected from the National Institute of Cardiology Outcomes Research (NICOR) database to obtain demographic and procedural details. Institutional review board approval was obtained by the Hospital’s Research Ethics board. All procedures which were performed over a 7-year period between July 2014 and March 2021 were included.

### Patient Selection

The decision to utilize a transfemoral approach for VSD closure in each patient was made following multi-disciplinary discussion at the joint cardiology/cardiothoracic conference.

Patients were included if they had a hemodynamically significant (symptomatic and/or left ventricular dilation, significant LV-RA shunt or progressive aortic regurgitation) PMVSD; weight ≤ 10 kg at time of procedure and the procedure was attempted via a transfemoral approach.

Patients were excluded if they had more than mild aortic regurgitation (AR), significant associated congenital heart disease, significant septal malalignment or aortic leaflet prolapse.

### Demographic Details

Data collected included demographic details (patient age, gender, weight, height, and body surface area at the time of the procedure); echocardiographic data (VSD measurement [LV entry, RV exit, VSD rims, presence or absence of aneurysmal tissue], associated anomalies, aortic, mitral or tricuspid valve regurgitation); and procedural data (sheath size, access type, angiographic VSD size, number of devices, associated procedures, residual shunting, fluoroscopic, procedural times, rhythm changes and reasons for failure if applicable).

Procedural success was defined by device implantation in the appropriate position without conversion to open heart surgery; which is our primary outcome. Secondary outcomes included any significant adverse events in relation to the procedure including death, rhythm disturbance, device migration, significant residual shunting (> 2 mm), requirement for pacemaker, or vascular access-related complication.

### Procedural Technique

**Fig. 1:**
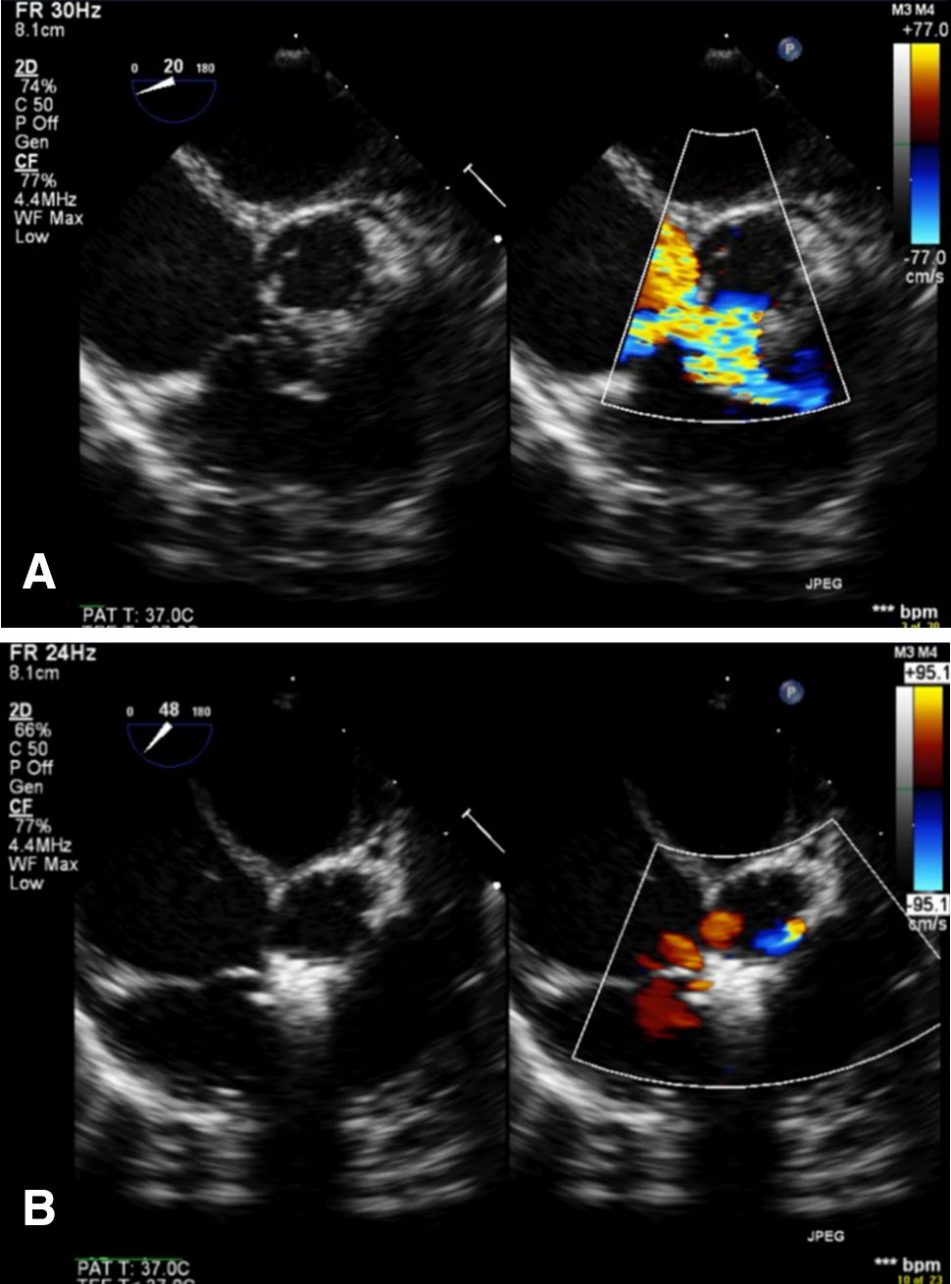
Transoesophageal echocardiography. **A** Demonstrates 2D and colour mode for a PMVSD with a moderate degree of tricuspid regurgitation pre-operatively. **B** Display the device in situ with mild degree of tricuspid regurgitation

Initially, transoesophageal echocardiogram (TOE) was performed under general anaesthesia to assess defect size, LV entry, RV exit, VSD rims, presence or absence of aneurysmal tissue and degree of valvular regurgitation, in order to determine suitability for device closure, and to aid in device selection (Fig. 1).

Patients were covered with periprocedural prophylactic antibiotics. Femoral arterial and venous access was obtained predominantly under ultrasound guidance after which 100 units/kg of IV Heparin was administered.

**Fig. 2:**
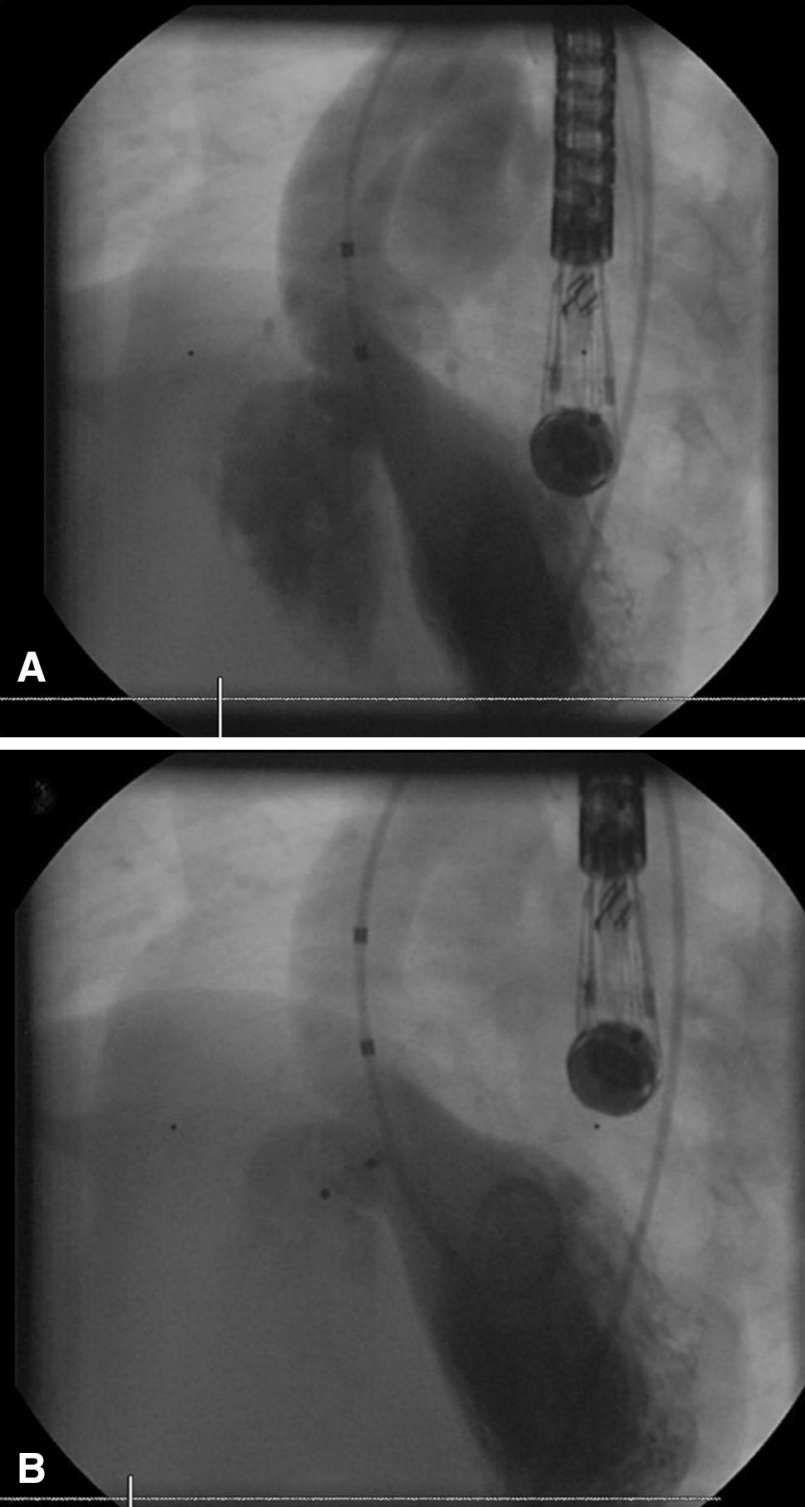
**A** Demonstrates left ventricular angiographic confirmation of the PMVSD in LAO 45 Cranial 25 degree view (Lower image). The lower image demonstrates final device position after device release demonstrating mild residual flow through the device

Left ventricular (LV) angiography was performed in a left anterior oblique view (Fig. 2).

The approach and the type of device was determined based on presence of a ventricular septal aneurysm, length of subaortic rim and VSD diameter with the retrograde (from the aorta) approach often initially preferred. The selected VSD device was chosen to be 1–2 mm greater than the largest TOE measurement of the VSD with colour flow. Once deployed, careful assessment of the interaction with the aortic and tricuspid valves was carried out with TOE. Cable induced aortic regurgitation and the inability to perform pre-release LV angiography have led to greater dependence on TOE imaging.

Post-procedure two additional doses of antibiotics were given every 8 h in the first 24 h. Aspirin was commenced at 5 mg/kg for 6 months. A comprehensive transthoracic echocardiogram and 12-lead ECG were performed on the day following the procedure.

### Statistical Analysis

Data were summarized as follows: continuous variables were summarized as median and interquartile range (IQR). Categorical data were summarized as count. Analyses were performed using Excel statistical function (Microsoft office standard 2013).

## Results

Sixteen patients underwent attempted transfemoral PMVSD device closure (12 retrograde) at a median age of 11 months (interquartile range [IQR] 9–15.5) and a median weight of 8.3 kg (IQR 7.2–9.5). Most patients were either symptomatic or had progressive left heart dilation. One patient had a significant LV-RA shunt with 3 + TR. Another patient had mild AR without aortic leaflet prolapse thought be related to the VSD. Median defect size on pre-procedural TOE was 6.8 mm (IQR 6–8.5). Median device waist size was 6 mm (IQR 4.5–8).

Genetic abnormalities were present in three patients, two of whom had 22q11 microdeletion and one with Down syndrome (Table 1).

**Table 1:** Demographic and pre-catheterization clinical details for the study group

Demographics	Study number
Total patients	16
Age, m (IQR)	11(9–15.5)
Weight, kg (IQR)	8.3 (7.2–9.5)
Body surface area (IQR)	0.39 (0.35–0.43)
Female, number (%)	6 (37.5)
Size of VSD, mm (IQR)	6.8 (5.7–8.3)
LVEDD, mm (IQR)	29 (28.3–31)
Z score (IQR)	1.85 (0.98–2.18)
Co-morbidity	
Down syndrome	1
22q11 microdeletion	2

### Procedural Outcomes

Six patients received Amplatzer Duct Occluder II (Abbott, Clonmel, Tipperary, Ireland); 4 patients received Lifetech Symmetric Membranous VSD Occluder (Lifetech, Shenzhen, China); 4 patients received KONAR-MF VSD Occluder (Lifetech, Shenzhen, China); 1 patient received Lifetech Eccentric Membranous VSD Occluder (Lifetech, Shenzhen, China); and 1 patient received Occlutech PmVSD Occluder (Occlutech, Helsingborg, Sweden). Median procedural and fluoroscopy times were 81 min and 12 min, respectively.

Median (IQR) device waist size was 6.2 mm (5.7–8.3 mm) (Table 2). Successful device placement was achieved in 14 patients (88%). Two patients required two devices due to significant residual shunt after release of the first device into a fenestrated defect (retrograde approach in both). Of the two unsuccessful device placements, one patient developed moderate aortic valve regurgitation (AR) and tricuspid valve regurgitation (TR) upon retrograde and antegrade device deployment, respectively. The device was not released and the patient was subsequently sent for elective surgical closure. The other patient developed progressive moderate AR 2 days post procedure, and the patient underwent surgical removal with no residual AR. One further patient developed transient haemolysis which resolved spontaneously within 1 week. There were no cases of device embolization and no other procedural complications, including no femoral arterial compromise. None of the patients required emergency surgery.

**Table 2:** Procedural and outcome details for the study group

Procedural and outcome details	Study number
Successful device deployment, *n* (%)	14 (87.5)
Anaesthesia used General anaesthesia, *n* (%)	16 (100)
VSD diameter, mm	6.8(6–8.5)
VSD device diameters	6.2(5.7–8.3)
Type of VSD device	
Amplatzer Duct Occluder II	6
Lifetech Symmetric Membranous VSD Occluder	4
KONAR-MF VSD Occluder	4
Lifetech eccentric membranous VSD occluder	1
Occlutech PmVSD occluder	1
Number of devices/patients	
Number of patient with (1/patient)	12
Number of patient with (2/patient)	2
Approach	
Retrograde	14
Antegrade	4
Procedural time (min)	81(50–95)
Fluoroscopy time (min)	12(8–17)
Length of hospital stay (days)	1 (1–1)
Follow-up duration (months)	40.5 (25–64)

Median hospital stay was 1 day (IQR 1–1). On median follow-up of 40.5 months (IQR 25–64), none of the patients developed complete heart block. Three patients (18.75%) had small residual shunts at latest follow-up which have not required any further intervention.

## Discussion

Since the initial report of transcatheter VSD closure by Lock et al. [16], the advent of transcatheter closure has progressed rapidly, initially with muscular VSD closure [17–19] and subsequently with PMVSD closure [20]. Early large series reported the challenges faced when dealing with infants weighing less than 10 kgs [13]. With this single centre experience, we report good success rates (88%) with device closure of PMVSD’s in this challenging cohort of patients with a median weight of 8kgs and defect size of 6 mm.

Often, the most challenging aspect of these cases in these smaller patients is pre-determination of device interaction with the tricuspid or aortic valves prior to release. This is particularly challenging in PMVSD’s where the aortic rim is deficient leading to potential impingement on the aortic valve resulting in significant aortic regurgitation [21–23]. Long-term rates of aortic regurgitation (AR) are unclear however a recent presentation evaluating 10-year follow-up with the original Amplatzer membranous occluder in 95 patients, demonstrated mild stable AR in 3 patients [24]. Significant aortic regurgitation requiring surgical removal has been reported at 1.8% [25]. AR may also occur in the absence of leaflet impingement and some authors have suggested distortion of the aortic annulus or subaortic membrane configuration with larger devices or by repeated crossing of the aortic valve [26]. It has also been suggested that when trivial or mild, the degree of AR many remain stable or decrease over time [26]. The improvement of AR is thought to be likely secondary to normalization of valve configuration changes created by repeated valve crossing or by change in configuration of the thin-walled membranous septum which improves with time [26]. It may be challenging to distinguish leaflet impingement from cable induced aortic distortion when performing these cases via the retrograde arterial approach and careful echocardiographic evaluation is essential to differentiate these two possibilities.

In this series the aortic valve was assessed closely with echocardiography upon crossing the VSD with the sheath, before device deployment and then before device release. Furthermore, ascending aortography was sometimes performed to complement echocardiographic images and delineate the extent, if any, of the AR. Although interpretation of aortography with retrograde delivery was less helpful, when performed through the side arm of the delivery sheath, this usually provided further information regarding potential device interaction with the valve leaflets. Multiple evaluations with echocardiography at different time points are important to understand the exact mechanism of AR and comparative evaluations once the sheath has been advanced into the RV and following device deployment can be useful in differentiating delivery cable from device related regurgitation.

Despite detailed assessment with complimentary imaging, two patients developed AR. The first patient developed moderate AR upon retrograde device deployment. The pre-procedural echocardiogram demonstrated an associated septal aneurysm, which has been shown to reduce the risk of AR [13]. On repositioning of the device using the antegrade approach the patient subsequently developed significant tricuspid regurgitation (TR). The device was recaptured and the patient was referred for surgery. In the second patient, who was noted to have a small aortic rim measuring < 2 mm, the device was deployed successfully initially with minimal AR. However, the patient developed progressive AR 2 days post procedure, and subsequently underwent surgical removal with no residual AR. Surgical examination intra-operatively revealed an intact aortic valve with no signs of aortic leaflet damage These two cases demonstrate the importance of a thorough assessment not only prior and during device deployment, but also in the early post-procedural period. They also highlight the importance of outlining the risk of valve distortion when consenting patients or their guardians.

Fourteen of the devices were implanted via the retrograde approach. In comparison to the antegrade approach, this approach involves comparatively fewer steps and carries the potential to significantly reduce fluoroscopy and total procedural times [27]. Avoidance of arteriovenous looping may also avoid cardiovascular compromise created by splinting of the heart. As has been reported in previous series, the avoidance of the arteriovenous loop is particularly important in small hearts as the wire and sheath crossing the tricuspid and aortic valves may cause severe haemodynamic compromise [23].

As most of the patients had an aneurysmal pouch, all but 1 patient underwent symmetric double-disc occluders. Only one eccentric membranous occluder was used in a patient where the aortic rim was incomplete.

**Fig. 3:**
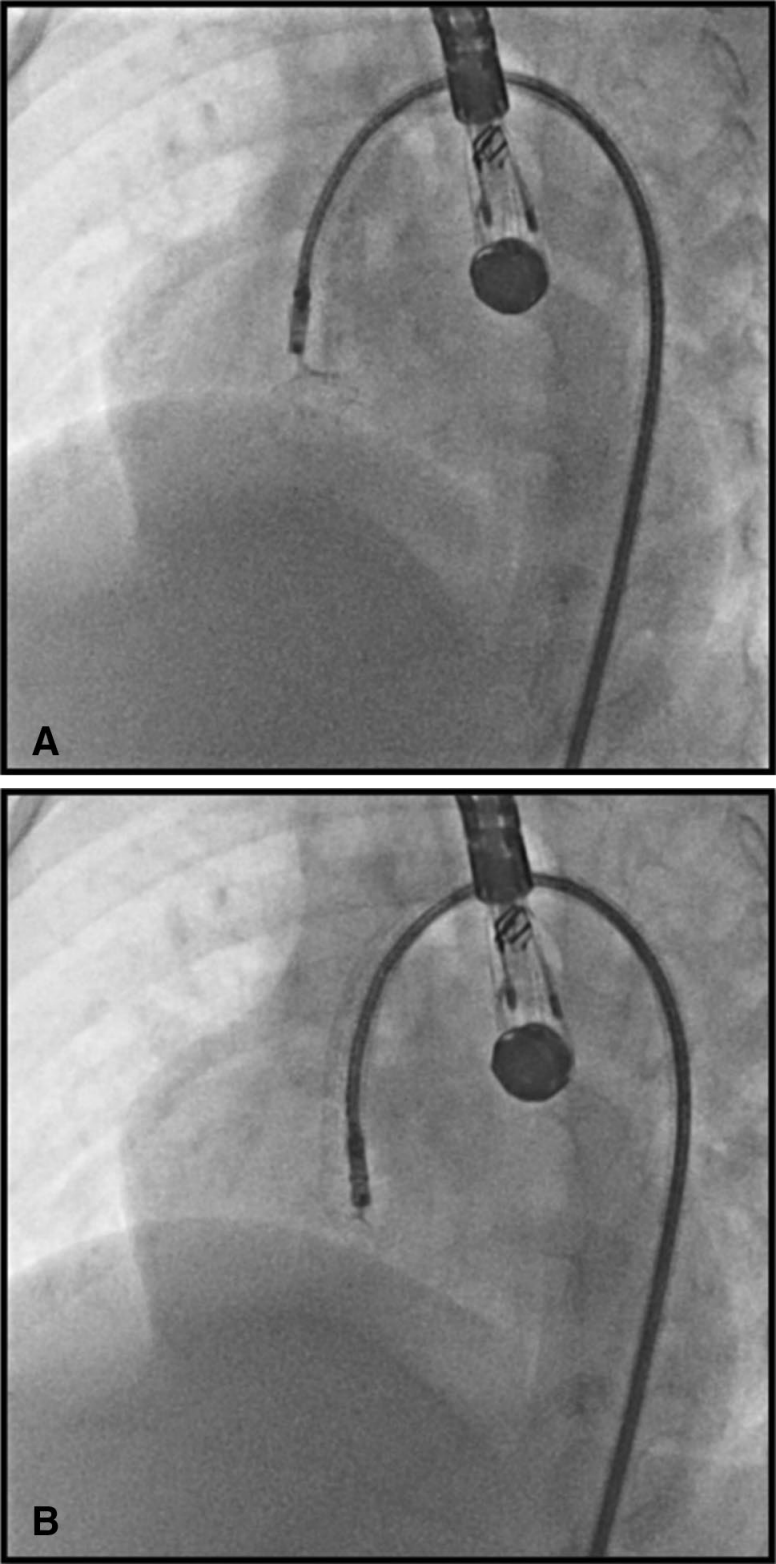
**A** Shows traction on the VSD device without curve on the cable around the aorta. **B** Better alignment of the cable to the device following manual curving of the cable with less traction on the VSD device

There have been concerns about large sheaths and delivery systems being used in the femoral arteries of these small children. However, the relatively large delivery systems used in the femoral artery in this series were well tolerated and none of the patients who underwent retrograde closure suffered femoral arterial compromise. Occasionally, modification of the delivery cable is required to minimize traction on the deployed LV disc caused by a straight delivery cable around the relatively acute natural curves of the aortic arch in a small heart (Fig. 3).

One of the less well considered benefits of the retrograde approach is the avoidance of delivery sheath disruption of the tricuspid valve (TV). However, it is important when using the retrograde approach to consider deploying the TV disc only when the support mechanism of the TV has been cleared, usually best seen using live TOE.

Mechanisms leading to tricuspid regurgitation (TR) with PMVSD closure include chordae tendinea injury or improper placement or oversizing of the device [28]. The exact anatomical variations in the relationship of the TV to the VSD that can predict procedural failure due to new onset TR have been challenging to identify. Certainly, cases where the defect extends up to the base of the septal leaflet of the TV may be more susceptible to device related distortion of the septal leaflet. This is likely to be relevant for those devices with larger “RV” discs including the ADO II. The incidence of TR with the ADO II is estimated to be 0.4 to 8% [26], 29–32. As the device is not particularly rigid the degree of TR in older patients is mostly mild and improves with time [26, 31]. Pre-existing TV regurgitation, which may be considered another indication to close the VSD, is reported to improve with PMVSD device closure. The mechanism is likely to be related to the commonly associated LV-RA shunt [26, 31, 33, 34]. One of our patients had pre-existing moderate to severe tricuspid regurgitation that decreased to mild degree after device closure.

While we favour the retrograde approach and it has the aforementioned benefits, flexibility during the case is important. While using the antegrade approach, it’s important to cross the TV using a balloon tipped catheter thus reducing the risk of TV chordae injury. The relationship between any aneurysmal tissue and tricuspid valve leaflet is an important consideration as a TV with a redundant leaflet may appear to be part of an aneurysm i.e. pseudoaneurysm but this is not a distinct true aneurysm leading to the device disc clamping on the TV chordae [31, 35].

Narin et al. reported transcatheter VSD closure in 12 patients less than 1 year of age via a transfemoral approach [36]. Eight were PMVSDs and 6 of these were performed via an antegrade approach. The smallest patient’s weight in their group was 4.8 kg. The choice of approach is largely dependent on the presence or absence of adequate aortic rim, presence of a VSD aneurysm and operator experience. In our centre, the initial attempt is generally via a retrograde approach, especially in the presence of a sufficient aortic rim and/or VSD aneurysm, with flexibility to revert to the transvenous approach if necessary.

While valvular regurgitation is a concern with transcatheter PMVSD closure, atrio-ventricular (AV) block has historically been the most feared complication [11]. In our series no patient in the follow-up period developed either transient or permanent AV block. A large meta-analysis has reported a rate of complete AV block of 1.1% with very few patients requiring permanent pacemaker insertion [11]. This was a heterogenous group of studies, and the age and weight of patients were not considered due to inconsistent reporting in the evaluated studies. Similar to this meta-analysis, we demonstrated excellent results when considering potential AV block complications.

## Conclusion

This series demonstrates that device closure of PMVSD’s in children weighing ≤ 10 kg is feasible and safe with good procedural success rates. Understanding the importance of complimentary imaging along with flexibility with approach ensures success with limited complications. Ultimately longer-term follow-up data are required as concerns still exist in relation to the possible interaction of the device in particularly with the aortic valve.

## Data Availability

Data available on request from the authors.
